# Management of Forgotten Groups after an Earthquake: Care Services for Drug Addicts

**DOI:** 10.18502/ijph.v49i8.3913

**Published:** 2020-08

**Authors:** Behzad HEYDARPOUR, Sahar NAJAFI, Maryam JANATMAKAN, Saeid KOMASI

**Affiliations:** 1. Cardiac Rehabilitation Center, Imam Ali Hospital, Kermanshah University of Medical Sciences, Kermanshah, Iran; 2. Lifestyle Modification Research Center, Imam Reza Hospital, Kermanshah University of Medical Sciences, Kermanshah, Iran; 3. Clinical Research Development Center, Imam Reza Hospital, Kermanshah University of Medical Sciences, Kermanshah, Iran

## Dear Editor-in-Chief

Natural disasters such as floods, storms, and earthquakes as inevitable events sometimes threaten the physical and mental health of various communities around the world (1). If severe, these misfortunes can lead to the death of people and the destruction of residential areas in cities and villages. After the disaster, the survivors of the catastrophe have been shocked and tortured due to the deaths of their relatives for several months. Among the bereaved, certain groups generally encounter more compatibility issues. Children, elderly, physically and mentally handicapped, and drug and alcohol addicts are among the groups (2). These people are often incapable of crisis management and require an effective support system (3). Unlike children, the elderly, and handicaps, generally considered to be officially supported, after a natural disaster, drug addicts are considered to be a forgotten group.

Obviously, drug addiction is an international health crisis that is common in both developed and developing societies (4). In normal conditions, addicts have routine access to illegal drugs and/or drug therapy and psychotherapy systems (3). Conversely, in times of crisis such as disasters, this access is faced with a serious challenge (2, 5). Immediately after a disaster like an earthquake, addicts are faced with physical symptoms and psychological crisis and are seeking illegal drugs. As well, patients undergoing treatment due to the destruction of treatment centers and crowds in medical centers are not able to provide the legal drug. Meanwhile, their informal support system does not have the same performance (3). Therefore, both groups are nervous and will likely be at high-risk behaviors and devices. This situation may facilitate the onset of fatal infections (6). Persons undergoing treatment may relapse and may also cause insecurity for others in shelters.

Based on these considerations and given the prevalence of drug addiction, especially in developing countries (4), the field specialists and policymakers should manage this vulnerable population in natural disasters (2, 5). Early delivery of medication and psychotherapy to the patients under treatment and the provision of syringes and safety devices for addicts use could potentially reduce future comorbidities and outbreaks in the population.
